# Effectiveness, safety, and economic evaluation of topical application of a herbal ointment, *Jaungo*, for radiation dermatitis after breast conserving surgery in patients with breast cancer (GREEN study): Study protocol for a randomized controlled trial

**DOI:** 10.1097/MD.0000000000015174

**Published:** 2019-04-12

**Authors:** Seungwon Shin, Bo-Hyoung Jang, Hae Sun Suh, Seung-Hyeok Park, Jin-Wook Lee, Seong Woo Yoon, Moonkyoo Kong, Yu Jin Lim, Deok-Sang Hwang

**Affiliations:** a Senior Researcher, Clinical Trial Center, Korean Medicine Hospital, Kyung Hee University, Seoul; b Assistant Professor, Department of Preventive Medicine, College of Korean Medicine, Kyung Hee University, Seoul; c Associate Professor, Pharmaceutical Economics, Outcomes Research and Policy, College of Pharmacy, Pusan National University, Busan; d Department of Clinical Korean Medicine, Graduate School, Kyung Hee University, Seoul; e Department of Clinical Korean Medicine, Graduate School, Kyung Hee University, Seoul; f Professor, Department of Korean Internal Medicine, Korean Medicine Cancer Center, Kyung Hee University Hospital at Gangdong, College of Korean Medicine, Kyung Hee University, Seoul; g Assistant Professor, Department of Radiation Oncology, Kyung Hee University Medical Center, Kyung Hee University School of Medicine, Seoul; h Clinical Assistant Professor, Department of Radiation Oncology, Kyung Hee University Medical Center, Kyung Hee University, 23 Kyungheedae-ro, Dongdaemun-gu, Seoul; i Associate Professor, Department of Korean Obstetrics and Gynecology, College of Korean Medicine, Kyung Hee University, 26 Kyungheedae-ro, Dongdaemun-gu, Seoul, Republic of Korea.

**Keywords:** breast neoplasms, herbal medicine, ointments, radiodermatitis, randomized controlled trial, traditional Korean medicine

## Abstract

**Introduction:**

This is a prospective, open-label, parallel-group, randomized controlled trial that evaluates the effectiveness and safety of adjuvant application of *Jaungo* (JUG) for radiation-induced dermatitis (RD) in breast cancer patients undergoing radiation therapy, in comparison with general supportive care (GSC).

**Methods/design:**

Eighty female patients, who have been diagnosed with unilateral breast cancer, will be allocated to either the JUG or GSC group with an allocation ratio of 1:1 after breast conservation surgery, in the Kyung Hee University Korean Medicine Hospital, Seoul, Republic of Korea. Both the groups will be subjected to GSC, but only the JUG group participants will apply adjuvant JUG ointment on the irradiated skin for 6 weeks, twice a day. The primary outcome of this study is the assessment of incidence rate of RD using the Radiation Therapy Oncology Group (RTOG) for toxicity gradation of 2 or more. Maximum pain level, quality of life, adverse reactions, and pharmacoeconomic evaluations will also be included.

**Discussion:**

The primary outcome will be statistically compared using the logrank test after estimating the survival curve using the Kaplan–Meier method. Continuous variables will be tested using independent *t* test or Mann–Whitney *U* test. The adverse events will be evaluated with Chi-square or Fisher exact test. All the data will be analyzed at a significance level of 0.05 (two-sided) with R software (The R Foundation).

**Trial registration:**

CRIS (Clinical Research Information Service), KCT0003506, 14 February 2019.

## 1 Introduction

Almost 90% of the patients undergoing radiotherapy for breast cancer have been reported to develop radiation-induced dermatitis (RD).^[1]^ The complaints vary from erythema to moist or dry desquamation^[2]^ that results in significant discomfort, restriction of daily activities, and even cessation of necessary radiotherapy.^[1]^ There have been several studies reporting prevention or treatment of RD with the external application of skin products including aloe vera gel,^[3]^ anionic phospholipid-based cream,^[4]^ wound healing ointment,^[5]^ corticosteroid therapy,^[6]^ or hyaluronic acid-based formulation.^[7]^ Breast intensity-modulated radiation therapy (RT) was also proposed to reduce the occurrence of moist desquamation in acute RD.^[8]^ Additionally, washing skin with water and soap during radiotherapy for breast cancer could be helpful because it could remove skin microbes that cause skin inflammation.^[9]^ However, there is no standard therapy or consensus on the optimal management of RD in the breast cancer patients till date.^[10]^

*Jaungo* (JUG) is a herbal ointment consisting of *Angelica gigas radix* and *Lithospermum radix*, which are the usual ingredients prescribed for various skin disorders in clinical practice of traditional Korean medicine (TKM), such as burns, eczema, psoriasis, or frostbite.^[11]^ There have been several in vivo and in vitro studies demonstrating the efficacy of JUG in dermatopathy. JUG has known to significantly promote wound healing on both the sterilized and infected wounds of Sprague-Dawley rats.^[12,13]^ Shikonin, the active compound of *Lithospermum radix*, enhances cell migration of intestinal epithelial cells through a mechanism that involves transforming growth factor-β induction, thus implying that JUG could help to heal intestinal injuries.^[14]^ Shikonin also significantly attenuates the inflammatory response in cellular models exposed to lipopolysaccharide.^[15]^ Furthermore, decursinol angelate, a coumarin compound of *Angelica gigas radix*, showed anti-inflammatory activities that inhibited the expression of pro-inflammatory cytokines and matrix metalloproteinase-9.^[16,17]^

There have also been a few clinical trials that explored the effectiveness and safety of JUG in RD. Patients undergoing RT for brain tumors showed favorable improvement or meaningful prevention of scalp dermatitis after topical application of JUG.^[18,19]^ A pilot study randomly allocated 30 breast cancer patients and evaluated the incidence of RD with Radiation Therapy Oncology Group (RTOG) toxicity of grade 2 or more, resulting in lowering of the RD incidence in the JUG treatment group with no safety issues, as compared to the general supportive care (GSC) group.^[20]^

This study protocol is primarily aimed at showing the effectiveness and safety of the adjuvant application of JUG for RD in breast cancer patients undergoing RT, as compared to GSC, along with its pharmacoeconomic evaluation.

## 2 Methods/design

This study protocol adheres to Standard Protocol Items: Recommendations for Interventional Trials (SPIRIT) 2013 statement,^[21]^ and Standard Protocol Items for Clinical Trials with Traditional Chinese Medicine 2018: Recommendations, Explanation and Elaboration (SPIRIT-TCM Extension 2018).^[22]^

### 2.1 Objectives and hypothesis

The primary objective of this study is to evaluate the effectiveness of adjuvant application of JUG on the irradiated skin of breast cancer patients undergoing RT after breast conserving surgery, with comparison of only GSC treatment. This will be assessed by the incidence rate of RD with RTOG toxicity grade of 2 or more. The secondary outcomes to be assessed and compared between the JUG and GSC arms include the time of initial RD occurrence, RD duration, maximum pain level, and quality of life (QoL). Additionally, the safety and pharmacoeconomic variables will be evaluated for JUG application in breast cancer patients.

### 2.2 Study design and setting

This is a prospective, open-label, parallel-group, randomized controlled trial. This study will enroll 80 female patients, who have been diagnosed with unilateral breast cancer confirmed by biopsy and supposed to undergo RT of 50 to 64 Gy dose after breast conserving surgery. They will be allocated to either the JUG or GSC group with an allocation ratio of 1:1. Both groups will be subjected to GSC, but only the JUG group will apply the adjuvant JUG ointment on the irradiated skin for 6 weeks, twice a day. Eligible participants will be enrolled from the in-patients or out-patients of the Kyung Hee University Korean Medicine Hospital, Seoul, Republic of Korea. Study advertisements will be posted on webpages and notice boards to promote subject enrollments.

**Figure 1:**
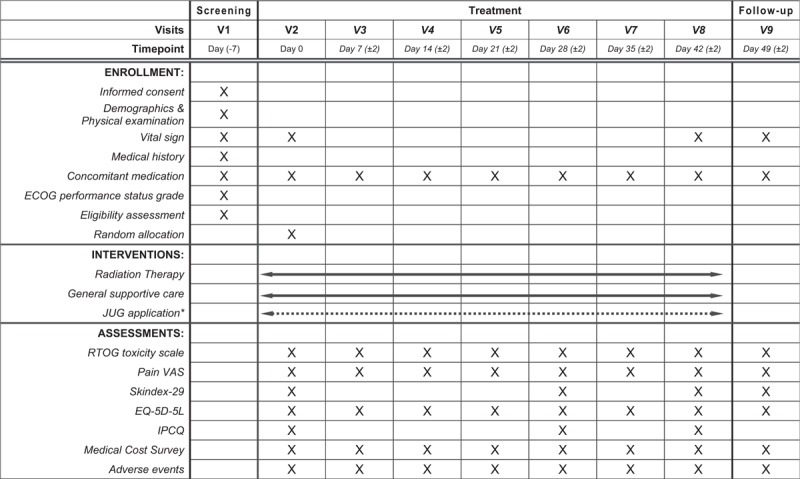
Study flow. ^∗^JUG treatment will be applied only for the intervention group (a dotted line). ECOG = Eastern Cooperative Oncology Group, EQ-5D-5L = EuroQoL Five Dimensions questionnaire 5-Level, IPCQ = iMTA Productivity Cost Questionnaire, JUG = *Jaungo*, RTOG = Radiation Therapy Oncology Group, VAS = Visual Analogue Scale.

The study flow is depicted in Figure 1.

### 2.3 Eligibility criteria

Female patients aged 19 to 80 years old and diagnosed with unilateral breast cancer based on biopsy are eligible for participation in this study. The other inclusion criteria include: RT with a total dose of 50 to 64 Gy without bolus, after breast conserving surgery; and Eastern Cooperative Oncology Group performance status of 1 grade or less. All the eligible participants have to provide voluntarily signed written/informed consent forms after understanding the trial details.

The exclusion criteria are: inflammatory breast cancer; skin invasion or distant metastasis of tumor; concurrent chemotherapy with RT, except for herceptin and hormone therapy; untreated wounds, severe/extensive burn, dampness, erosion, or suppuration on breast skin; fever; history of prior RT to the chest wall; history of connective tissue disorders; history of heart diseases such as arrhythmia, uncontrolled hypertension, severe mental disorders (dementia, drug addiction, etc.) demanding intensive treatment, or paralysis; or history of hypersensitivity reaction to the study drug or it is ingredients. Participants who are involved in other trials within that month or disagree with contraception during the study period will also be considered ineligible.

### 2.4 Randomization, allocation concealment, and blinding

An independent statistician will generate random sequence numbers using the R version 3.5.2 (The R Foundation) with blockrand package version 1.3. Eligible participants will be randomly allocated to either the JUG or GSC group in a ratio of 1:1, and the allocation will be concealed using the sealed envelope method. The sealed envelopes will include random numbers and allocated groups, and the investigators will open the envelopes to assign the participants to particular groups after completing the screening process. Since JUG will be prescribed only to participants of the JUG treatment group, this trial is an open-label study.

### 2.5 *Jaungo* treatment

JUG ointment (product name: *Hanpoong Jaungo*, pharmaceutical company: *Hanpoong Pharm*. Co Ltd, Seoul, Republic of Korea) consists of two active herbs, *Angelicae gigantis radix* (*Angelica gigas Nakai*, family Umbelliferae) 60.6 mg/g and *Lithospermi radix* (*Angelica gigas Nakai*, family Boraginaceae) 72.7 mg/g, which are standardized based on the Korean Pharmacopoeia. The ointment also contains sesame seed oil, bees wax, and swine oil as carriers. JUG is packaged as a 15 g pumping bottle. The participants assigned to the JUG group will be provided with two bottles of JUG on the 2nd (day 0) and 5th visit (day 21), respectively. The JUG has to be applied on the irradiated parts of the breast skin by themselves, twice a day (every morning and evening) for 42 days, which is the period of RT for breast cancer. Patients are supposed to record drug use diary and return the bottles on the next visit for adherence evaluation.

### 2.6 Radiation therapy and general supportive care

All the patients will undergo RT at a dose of 2 Gy/day without bolus by a medical specialist of radiation oncology. The whole breast irradiation with photon beam will be implemented for 5 weeks (5 times a week) and a boost irradiation with photon or electron beam will be added for 1 week (5–7 times a week), if necessary. Additionally, the regional lymph node irradiation could be included, covering axilla level II/III for irradiation.

Participants allocated to the treatment or control group will be instructed to wash the irradiated skin with clean water or wet towels, and wipe the skin with dry towels in order to keep the irradiated skin clean and dry. Furthermore, any lotion or cream can be applied to the breast skin. This GSC regimen is based on a preceding study^[9]^ and a pilot trial.^[20]^

Any other type of preventive or treatment management of RD is prohibited during study participation.

### 2.7 Outcomes

Data will be collected and recorded in case report forms. An independent monitor will regularly visit the study site for source data verification, investigator product management, protocol deviation check, and safety issue monitoring.

### 2.8 RTOG toxicity scale

The primary outcome of this study is the incidence rate of RD of RTOG toxicity grade 2 or more. A radiation oncologist will assess the irradiated skin of the patients at every visit, based on the Cooperative Group Common Toxicity Criteria for skin suggested by the RTOG Foundation (https://www.rtog.org). The toxicity is graded at 6-levels: 0 for none or no change, 1 for scattered macular or papular eruption or erythema that is asymptomatic, 2 for scattered macular or papular eruption or erythema with pruritis or other associated symptoms, 3 for generalized symptomatic macular, papular, or vesicular eruption, and 4 for exfoliative dermatitis or ulcerating dermatitis.

Furthermore, the time of RD onset and RD duration with grade 2 or more of RTOG toxicity will be evaluated.

### 2.9 Visual Analogue Scale (VAS) for maximum pain level

Patients will be questioned about the severity of their maximum pain level during RT procedures and will have to mark the pain level on a 10-cm line, where 0 represents “not painful at all” and 10 “extremely painful.”

### 2.10 Skindex-29

Skindex-29 is a patient-rated questionnaire to measure QoL of patients with skin diseases every 4 weeks, consisting of 30 items.^[23]^ It is a 5-level Likert scale from 1 (never) to 5 (always). The Korean version of Skindex-29 has been validated.^[24]^

### 2.11 Safety assessment

The adverse events will be evaluated and reported during the study. Local rash, rubescent, or itching are expected to be found from time to time. If an adverse reaction is found, then the investigators will assess the intensity/frequency of each episode and decide whether further treatments are necessary or not. Vital signs will be regularly checked to assess JUG safety.

### 2.12 Pharmacoeconomic evaluation

EuroQoL Five Dimensions questionnaire (EQ-5D) will be used to assess the pharmacoeconomic evaluation of JUG in breast cancer patients. The EQ-5D comprises 5-level questions on mobility, self-care, usual activities, pain/discomfort and anxiety/depression, and VAS. Quality adjusted life year (QALY) will be calculated with the EQ-5D results, which will be combined with medical cost survey to estimate the incremental cost-utility ratio and incremental cost-effectiveness ratio. We will use the validated Korean version of this outcome.^[25]^

The iMTA Productivity Cost Questionnaire (iPCQ) will assess the health-related productivity losses. This questionnaire has 12 items and the patients have to answer each by recalling the previous 4 weeks.^[26]^

### 2.13 Sample size

In the pilot study, occurrence rate of RD with RTOG toxicity grade 2 or more was 46.7% and 78.6% in the JUG and GSC groups, respectively.^[20]^ The null hypothesis is that *S*_*JUG*_ (t) = *S*_*GSC*_ (t), where *S* denotes the probability that RD does not occur during RT of breast cancer patients (*S*_*JUG*_ (t) = 53.3%, *S*_*GSC*_ = 21.4%). The sample size has been calculated as 36 per group (two-sided, α = 0.05 and 1-β = 0.8) with logrank test of PASS software version 14.0.11 (NCSS Statistical Software). With a 10% drop-out rate expected, we have decided to enroll 80 eligible participants (40 in each group).

### 2.14 Statistical analysis

The primary outcome is the incidence rate of RD graded 2 or more on the RTOG toxicity scale. These results will be statistically compared between groups using the logrank test after estimating the survival curve with Kaplan–Meier method. Any continuous variables will be tested independently with *t* test or Mann–Whitney *U* test according to the normality of the distribution. The adverse events will be tested with Chi-square or Fisher exact test. Paired *t*test or McNemar test will be adopted to compare the vital signs results within a group. All the data will be analyzed at a significance level of .05 (two-sided) with R software version 3.5.2 or later.

The primary analysis will include all the participants assessed with the RTOG toxicity scale at least once after randomization (full analysis set). Secondarily, per protocol set will be analyzed, which is defined as the group of participants complying with the trial procedure, including the study drug use, at least 80%. Safety set is defined as the participants who use the study drug at least once.

### 2.15 Ethics and dissemination

The study protocol and the informed consent form have been reviewed and approved by the Institutional Review Board in Kyung Hee University Korean Medicine Hospital, Republic of Korea (KOMCIRB-2018-10-003) on 14 December 2018 (Protocol V1.3) and registered in Clinical Research Information Service (CRIS, https://cris.nih.go.kr/cris/en/, KCT0003506) on 14 February 2019. Any amendments of protocol and consent forms will be valid only after being reviewed and approved by the Institutional Review Board in Kyung Hee University Korean Medicine Hospital and be publicized via CRIS.

Medical doctors will give the full information to any potential participant and obtain the written consent. Any data of the enrolled participants will be collected only with screening/random codes and their initials. Any personal information including identification code and their names will not be recorded in the case report forms nor shared with others.

The datasets used and/or analyzed after completing the current study will be available from the corresponding author under reasonable requests. The investigators will disseminate the study results and implications via publication.

## 3 Discussion

This is a prospective, open-label, parallel-group, randomized controlled trial to evaluate the effectiveness and safety of adjuvant application of JUG for RD in breast cancer patients undergoing RT, and compare with GSC. Eighty female patients with unilateral breast cancer after breast conserving surgery will be allocated to either JUG or GSC groups with an allocation ratio of 1:1. Both groups will undergo GSC, but only the JUG group will apply the adjuvant JUG ointment on the irradiated skin for 6 weeks, twice a day. Pharmacoeconomic evaluation of JUG will also be investigated.

JUG is a herbal ointment that has been authorized as a drug for xerosis cutis, frostbite, miliaria, anal fissure, and rhus dermatitis by the Ministry of Food and Drug Safety of the Republic of Korea.^[11]^ In clinical practice, many TKM doctors have used JUG for a variety of dermatopathy symptoms. With respect to the experimental evidences,^[12,13,15–17]^ we assumed that there could be a positive possibility to use JUG for RD. We have learned the effect size from the pilot study and expect significant results from this full-scale clinical trial.

## Author contributions

**Conceptualization:** Seungwon Shin, Yu Jin Lim, Deok-Sang Hwang.

**Data curation:** Seungwon Shin, Deok-Sang Hwang.

**Funding acquisition:** Deok-Sang Hwang.

**Investigation:** Bo-Hyoung Jang, Hae Sun Suh, Seung-Hyeok Park, Jin-Wook Lee, Seong Woo Yoon, Moonkyoo Kong, Yu Jin Lim, Deok-Sang Hwang.

**Methodology:** Seungwon Shin, Hae Sun Suh, Yu Jin Lim, Deok-Sang Hwang.

**Project administration:** Deok-Sang Hwang.

**Resources:** Bo-Hyoung Jang, Seong Woo Yoon, Moonkyoo Kong.

**Supervision:** Deok-Sang Hwang.

**Visualization:** Seungwon Shin.

**Writing – original draft:** Seungwon Shin.

**Writing – review & editing:** Seungwon Shin, Bo-Hyoung Jang, Hae Sun Suh, Seong Woo Yoon, Moonkyoo Kong, Yu Jin Lim, Deok-Sang Hwang.

Deok-Sang Hwang orcid: 0000-0001-9179-0797.
